# Efficacy of Oral Gabapentin and Acetaminophen for Postoperative Analgesia in Anorectal Surgery: A Fuzzy Logic Evaluation

**DOI:** 10.34172/mejdd.2024.378

**Published:** 2024-04-30

**Authors:** Seyed Jalal Ishagh Hosseini, Pouya Derakhshan Barjoei, Mojdeh Bahadorzadeh, Amin Seifaddini, Mostafa Vahedian

**Affiliations:** ^1^Department of General Surgery, Faculty of Medicine, Qom University of Medical Sciences, Qom, Iran; ^2^Department of Electrical Engineering, Naein Branch, Islamic Azad University, Naein, Iran; ^3^Department of Social and Family Medicine, Faculty of Medicine, Qom University of Medical Sciences, Qom, Iran

**Keywords:** Anorectal pain, Acetaminophen, Gabapentin, Anorectal surgery, Fuzzy logic

## Abstract

**Background:**

The present study attempted to evaluate the effect of oral gabapentin and acetaminophen for postoperative analgesia in anorectal surgery.

**Methods:**

This double-blind clinical trial was carried out on 144 patients who were candidates for anorectal surgery. The patients were randomly assigned into three groups of control, acetaminophen 500 mg, and gabapentin 300 mg for two hours before the surgery. Data on pain severity based on the visual analog scale (VAS) were evaluated and analyzed.

**Results:**

The results of the current study indicated that in patients taking acetaminophen and gabapentin tablets before surgery, the amount of postoperative pain decreased, and the amount of decrease in postoperative pain in the patients who received acetaminophen and gabapentin tablets compared with the placebo group was significant (*P*<0.001). Also, an evaluation was done using a proposed fuzzy logic model.

**Conclusion:**

Taking acetaminophen and gabapentin tablets one hour before the operation causes a significant reduction in postoperative pain in patients who are candidates for anorectal surgery. The results are promising and encourage one to pay attention to more studies with the goal of possibly using them as a decision-support model in the future.

## Introduction

 Anorectal surgery includes a wide range of surgeries, from hemorrhoidectomy to the internal anal sphincterotomy for rectal cancer.^1^ Perianal surgeries can be performed under general and spinal anesthesia. In recent years, different drugs and compounds have been used orally, intravenously, and intrathecally to improve the quality of analgesia. Proper control of postoperative pain leads to improved recovery, faster return to daily activities, faster discharge of patients, and reduced patient costs.^2^ Most pain control studies focus on postoperative drug regimens for pain control rather than prevention.^3,4^ However, this approach has not been done well for patients who undergo anorectal surgery.^5^ Of course, few studies have been conducted regarding the use of analgesics to prevent and control pain after colorectal surgeries. The results of this study have shown that postoperative pain control with acetaminophen, as a basic pain medication without the use of epidural anesthesia, is a painless, safe, and useful method.^6^ In a study that investigated the effects of oral tizanidine in preventing pain after anorectal surgery, the result of the study showed that the administration of oral tizanidine before anorectal surgery improves the severity of pain after surgery without increasing side effects.^7^ A study aimed at evaluating the effect of gabapentin on post-hemorrhoidectomy pain has shown that daily use of gabapentin in the period before surgery significantly reduces the reported amount of postoperative pain.^8^ Complications of gabapentin 300 mg tablets in a single dose are very rare.^8^ Gabapentin and acetaminophen have a positive effect on pain relief.^9,10^ Considering the high prevalence of anorectal surgeries and the importance of pain control in these patients, no study comparing the effectiveness of acetaminophen and gabapentin in controlling pain after anorectal surgeries has been performed. This study was conducted with the aim of comparing the effectiveness of two drugs, acetaminophen and gabapentin, in managing the pain of anorectal surgeries. Fuzzy logic is a soft computing method that has been developed to recreate the ability of the human mind to learn and make logical decisions in uncertain conditions.^11-13^ Applying fuzzy logic, the factors were converted into linguistic variables and were analyzed by the proposed fuzzy model.

## Materials and Methods

**Figure 1:**
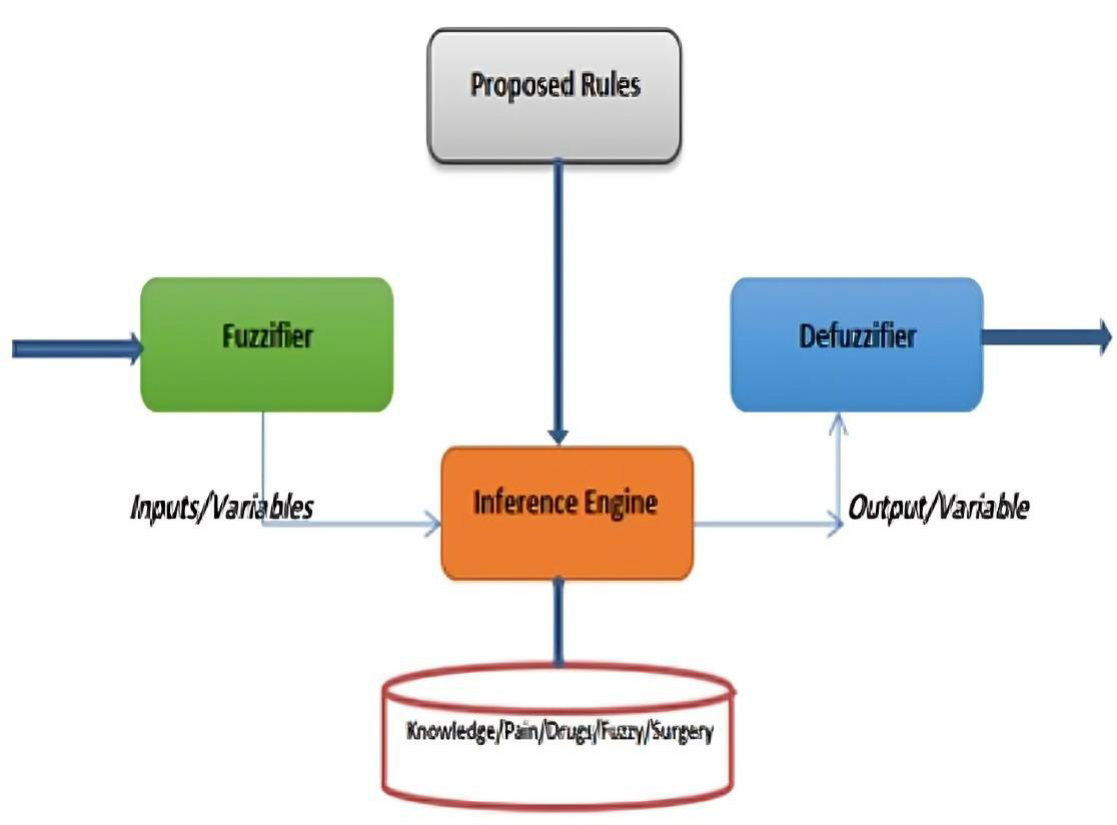
Scheme of fuzzy system

 After approval by the local ethics committee and obtaining written informed consent, in this double-blind, randomized clinical trial study, 144 patients with a mean age of 50.97 ± 5.24 years with anorectal surgery were recruited. The patients were randomly assigned to three groups of acetaminophen 500 mg tablets, gabapentin 300 mg tablets, and placebo. All three drugs were in the same shape and prescribed to the patients, two hours before the operation. Then, one hour after the operation, the patient’s pain level was measured using visual analog scale (VAS) pain scale. Patients’ demographic data, including sex and age, were initially recorded on a checklist. The surgeon and the researcher were blinded to the type of medications used for each patient. All patients underwent the same surgical procedure by the same surgeon. In the end, all data were analyzed using SPSS software, and a P value of less than 0.05 was considered statistically significant. By designing a fuzzy logic model, a model for decision-making and the impact of factors on pain in patients was investigated. By this algorithm, the decision structure was proposed by creating rules in the form of “if-then”. These rules were designed according to the knowledge of expert doctors. The structure of a fuzzy system includes a fuzzifier, inference engine, defuzzifier and knowledge database (Figure 1).

**Figure 2:**
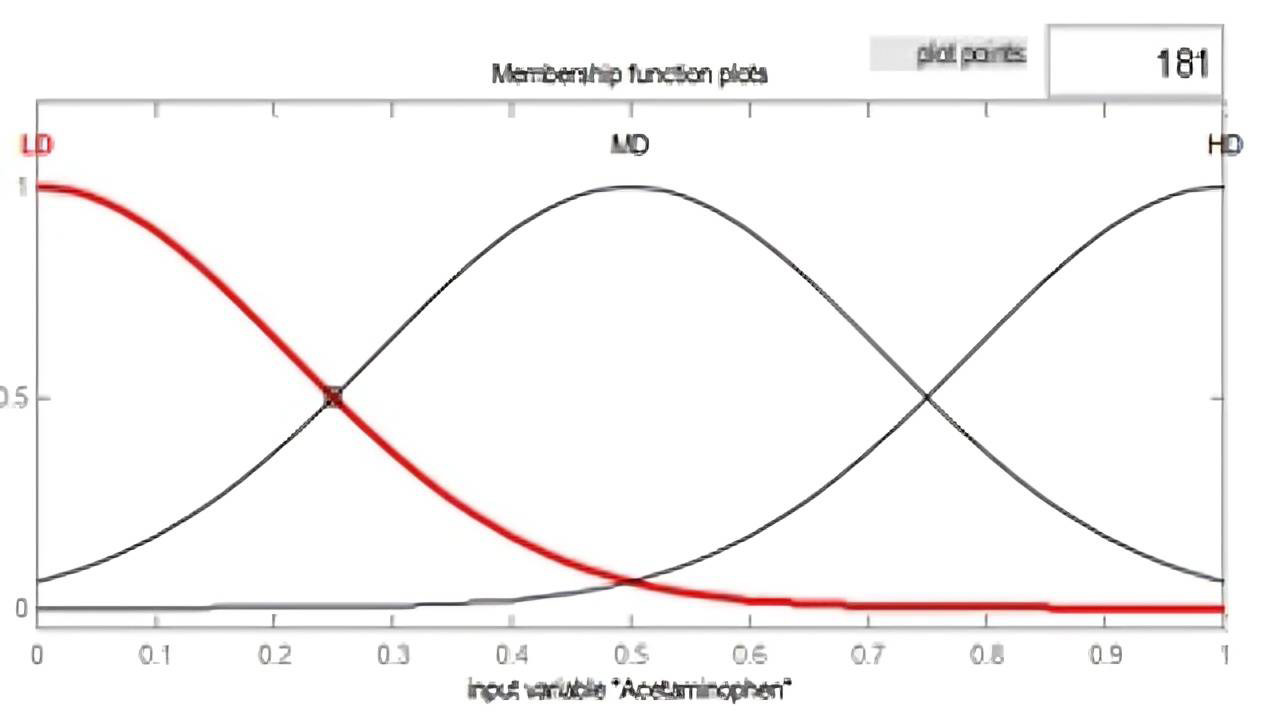
Membership function of input 1

**Figure 3:**
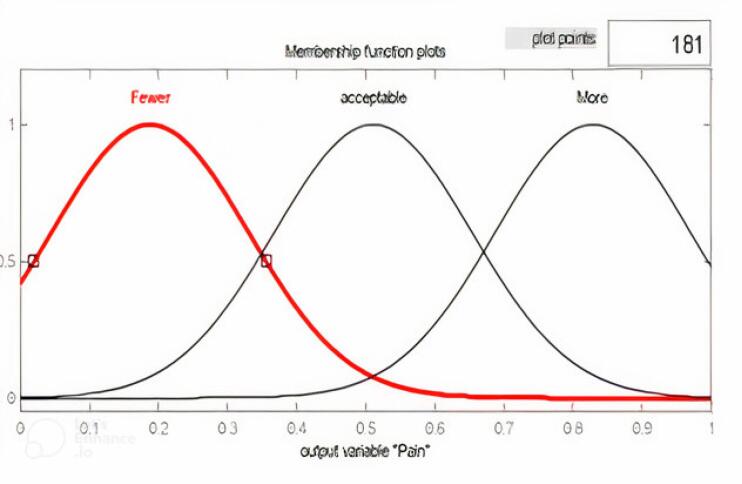
Membership function of pain

 In the fuzzifier section, the desired variables convert into linguistic values. In the second part, we specified the mechanism of fuzzy inferencing. In the defuzzifier, it is tried to convert the outputs from the fuzzy system into explicit values.^11-13^ The values and types of membership functions and fuzzy rules are set in the knowledge database section. Considering that the goal is to determine the relationship between factors affecting pain in patients, we modelled the effective variables according to the expert doctor’s diagnosis with a fuzzy system. Sample membership functions were chosen as Gaussian and based on the collected data. Gaussian membership function was also considered for outlet and pain. A typical membership function for one of the inputs as acetaminophen and output as pain are shown as follows (Figures 2 and 3).

 In the defuzzifier section, the proposed Mamdani fuzzy system with the minimum inference rule and the combination of fuzzy rules in the maximum-minimum form was designed. Fuzzy rules extracted from the proposed model were used in this system.

**Figure 4:**
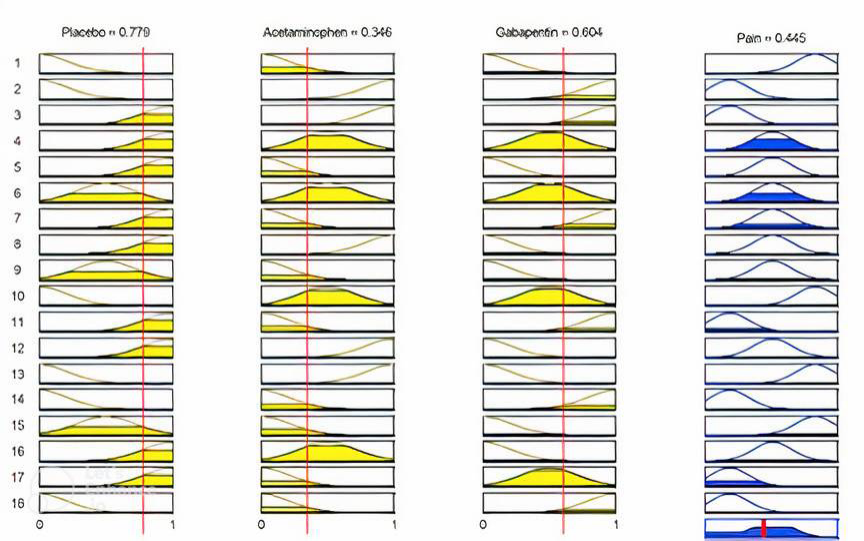
proposed fuzzy rules scheme

 These rules were designed according to the knowledge of expert doctors. The evaluation of the effects of factors is figured out, as shown in Figure 4, as a sample.

## Results

**Figure 5:**
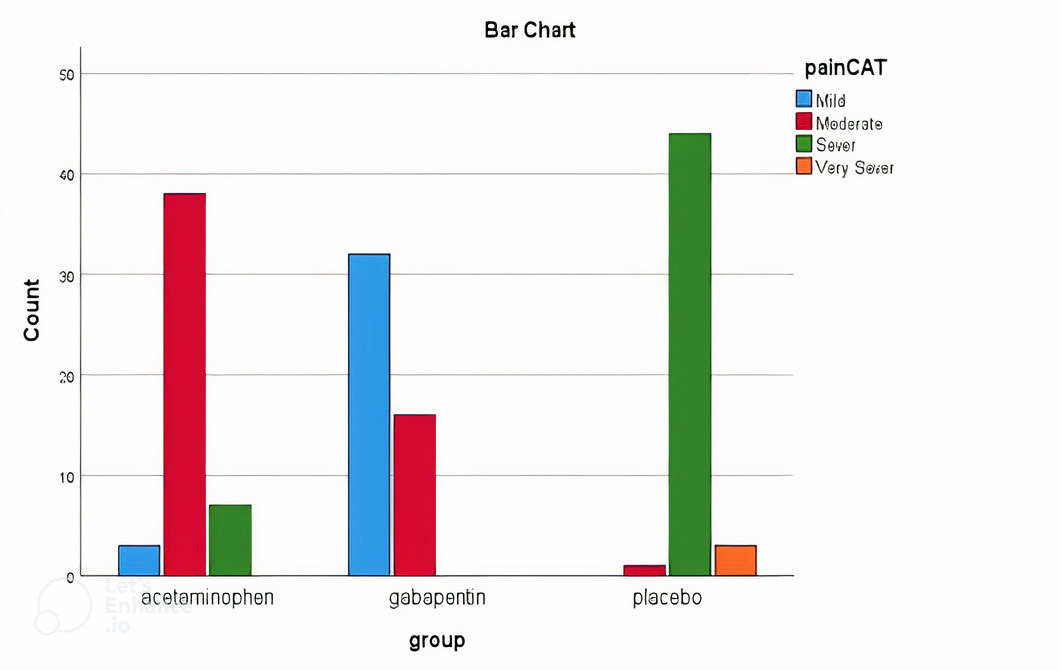
Comparison of pain intensity one hour after surgery

 The present study was performed on 144 patients from 2020 to 2022 with anorectal surgery. The patients were randomly assigned into three groups: control (placebo), acetaminophen, and gabapentin. There were no significant differences between the three groups in terms of mean age (*P* > 0.05), and sex (*P* = 0.92). The results showed a significant difference in pain score between the three groups (*P* < 0.001) one hour after surgery. The means of sedation scores showed a significant difference across all the three groups (*P* < 0.001). The results of comparisons showed a lower pain score one hour after the surgery in the acetaminophen group than in the placebo group. The average difference in pain measured one hour after the surgery was significantly different in the acetaminophen group and the placebo, and there was also a significant difference between the two groups of gabapentin and placebo (Figure 5).

 The average pain in the acetaminophen group compared with gabapentin was -2.10, which has a statistically significant difference (P*=*0.0001; Table 1).

**Table 1:** The average difference in pain measured

**Depend. Variable**	**Group (i)**	**Group (j)**	**Diff. Average (i-j)**	**Sig.**
Age	Acetaminophen	Gabapentin	-0.27	0.96
		Placebo	-0.54	0.87
	Gabapentin	Acetaminophen	0.27	0.96
		Placebo	-0.27	0.96
	Placebo	Acetaminophen	0.54	0.87
		Gabapentin	0.27	0.96
Pain	Acetaminophen	Gabapentin	2.10	0.00
		Placebo	-3.02	0.00
	Gabapentin	Acetaminophen	-2.10	0.00
		Placebo	-5.12	0.00
	Placebo	Acetaminophen	3.02	0.00
		Gabapentin	5.12	0.00

 Increasing age did not have a significant effect on reducing or increasing pain, although the volunteers in this study were close to each other in terms of age.

## Discussion

 This study investigated the efficacy of oral gabapentin and acetaminophen for postoperative analgesia in anorectal surgery. The population of this research included 144 patients who were candidates for anorectal surgery. They were eligible to enter the study, randomly and blindly divided into three groups of receiving oral acetaminophen 500 mg tablets, gabapentin 300 mg tablets, and placebo. Their pain levels were measured by VAS and numeric rating scale criteria. Each group included 48 people. Anorectal surgery represents different parts of the procedures in the general surgery department, which includes a wide range of surgeries from hemorrhoidectomy to internal anal sphincterectomy for rectal cancer.^1^ Lower limb and perianal surgeries can be performed under general and spinal anesthesia. In recent years, different drugs and compounds have been used orally, intravenously and intrathecally to improve the quality of analgesia. Proper control of postoperative pain leads to improved recovery, faster return to daily activities, faster discharge of patients, and reduced patient costs.^2^ Gabapentin and acetaminophen have a positive effect on pain relief. Masanori Naito and his colleagues conducted an intervention with the aim of pain management using acetaminophen in laparoscopic colorectal surgeries. The result of this study showed that postoperative pain control with acetaminophen as a basic analgesic drug, without the use of epidural anesthesia, is an acceptable method. It is easy, safe, and useful.^6^ In a study conducted in Iran by Rupaniet al, they investigated the effects of oral tizanidine in preventing pain after anorectal surgery. The results of their study showed that the administration of oral tizanidine before anorectal surgery reduced the severity of pain after surgery without increasing side effects.^7^ Poylin and colleagues conducted a study to evaluate the effect of gabapentin on pain after hemorrhoidectomy. They also concluded that daily use of gabapentin in the preoperative period significantly reduced postoperative pain.^8^ Acetaminophen did not have side effects in low doses.^6^ Complications of gabapentin 300 mg tablets in a single dose are very rare.^8^ Considering the high prevalence of anorectal surgeries and the importance of pain control in these patients, and as there is no study comparing the effectiveness of acetaminophen and gabapentin in controlling pain after anorectal surgeries, this study was conducted with the aim of comparing the effectiveness of two drugs, acetaminophen and gabapentin, in managing the pain of anorectal surgeries, which showed that taking acetaminophen 500 mg tablets and gabapentin 300 mg tablets before surgery reduced postoperative pain in patients. This reduction in pain compared with the placebo group was statistically significant. The results of the proposed fuzzy model showed a helpful scheme for decision-making. It is possible to design an inference model from the linguistic variables by determining the appropriate rules to investigate the effect and correlation of the factors on the desired variable, which is expected to be effective in helping to increase decision-making knowledge in the future.

## Conclusion

 The results of the present study proposed that the usage of acetaminophen and gabapentin two hours before the anorectal surgery significantly reduced the severity of pain after surgery. Hence, considering the effects of acetaminophen and gabapentin on pain relief after anorectal surgery and its minor side effects, these medications can be suggested as effective drugs to decrease pain after anorectal surgery. Fuzzy results also confirmed the effect of factors concordance with pain in patients.
